# Investigation on mechanical properties deterioration of concrete subjected to freeze–thaw cycles

**DOI:** 10.1038/s41598-022-27122-w

**Published:** 2022-12-30

**Authors:** Ruifeng Xie, Jianlin Yang, Enpu Xie

**Affiliations:** 1 School of Management Engineering, & Engineering Research Center Program of Development & Reform Commission of Jiangsu Province, Jiangsu Urban and Rural Construction Vocational College, Changzhou, 213147 People’s Republic of China; 2 Ocean and Civil Engineering, Shanghai Jiao Tong University, Shanghai, 200240 People’s Republic of China; 3 School of Civil Engineering, Huzhou Vocational and Technical College, Huzhou, 213147 People’s Republic of China

**Keywords:** Engineering, Materials science

## Abstract

Concrete structures in cold regions are usually suffer from froze and thaw action. A combined investigation of nanoindentation technique and X-ray diffraction were adopted to demonstrate the microstructure change and micromechanical properties deterioration of concrete subjected to freeze–thaw (F-T) cycles in this study. The results showed that the indentation modulus and hardness of the main compositions in mortar, such as calcium-silicate-hydrates and calcium hydroxide, both gradually decreases as the F–T cycles increase, with the greatest reduction approximate 38% after 1500 F–T cycles, while the corresponding greatest reduction of the main compositions in interfacial transition zone (ITZ) is close to 50%. In addition, the micropores in mortar and ITZ both gradually converge and connect to form larger diameter pores, and the thickness of ITZ increased rapidly from 25 to 50 μm after 1500 F–T cycles. On this basis, the effective modulus of elasticity under different F–T cycles are analyzed through Mori–Tanaka scheme with consistent variation tendency of dynamic modulus of elasticity test. Subsequently, the mechanical properties deterioration of concrete under F–T cycles is mainly attributed to the decrease of mechanical properties (such as modulus and hardness) of microscopic components, and the increase and propagation of the internal micropores especially for micropores in ITZ.

## Introduction

Freeze–thaw (F–T) action is one of the major causes for deterioration of durability performance of concrete structures in cold northern regions, such as bridge deck, dams and concrete ocean platforms etc. Many researchers have studied the effect and mechanism of F–T. The deterioration could be mainly attributed to hydraulic pressure, crystallization pressure and the mismatch of thermal effects between ice and solid phases^1–3^. So far, investigations on the deterioration of concrete mainly focus on the mechanical properties at macro-scales such as stress characteristic, tensile properties, mechanical properties of flexural structure members, and have revealed a reduction in the mechanical resistance^4–7^.

The macro-mechanical properties of composites are dependent on microstructure and micromechanical properties. As a typical composite material, concrete has complex microstructure and presents multi-scale characteristics. Numerous recent papers have attempted to investigate the freezing behavior through numerical simulation method, and to investigate the deterioration of cemented materials at mesoscopic scale based on above frost damage mechanism^8–10^. In addition, the microstructure changes of concrete under F–T action have often been investigated by means of X-ray diffraction (XRD), scanning electron microscope, ultrasonic imaging, X-ray computed tomography and so on^11–13^. Generally, the macromechannical performance of concrete presents opposite changes as the porosity increases when exposed to F–T cycles condition and ITZ crack width^14^. The macroscopic deformation and the effect on pore size distribution could be analyzed through the link of thermodynamics and poromechanics^15,16^. Correspondingly, some measures and various additives which could improve internal pore systems, and conceptual designs were proposed to enhance the macromechannical performance^17,18^. As for the micromechanical properties of concrete, nanoindentation technology could provide an effective method to detect the nanomechanical properties of micro components. During indentation test, the nanoscale indenter tip is pressed into the surface of tested material, force and displacement are recorded, and a load–displacement curve is generated subsequently. According to the curve, stiffness, hardness and indentation modulus can be derived by applying continuum scale models, respectively^19^. Some researchers have studied the indent modulus and hardness of main components of hardened cement paste using this technique^20,21^. Subsequently, the elastoplastic and creep properties of calcium-silicate-hydrates (C-S–H) gels, and the influence of anaphases materials addition on the properties of cemented materials were investigated by means of through this technique^22–24^.

The relation between macromechannical performance and microstructure and micromechanical properties is relatively significant. Under the F–T cycles, the microstructure of concrete and the micromechanical properties of individual component could be changed. However, the corresponding researches of are indeed of scarce. Moreover, it is extremely important to study the changes of microstructure and micromechanical properties under F–T cycles for more in-depth understanding of the reduction mechanism of macroscopic performance. Therefore, in this study, a combination of nanoindentation and XRD is utilized to conduct a comprehensive investigation on the microstructure and mechanical properties of concrete subjected to F–T cycles. Firstly, the micromechanical properties of individual component of mortar, interface transition zone (ITZ) subjected to different F–T cycles were investigated. Secondly, through the analysis of the distribution and expansion of micropores, the thickness of ITZ was characterized. Finally, the effective modulus of elasticity of concrete under different F–T cycles are analyzed by means of homogenization theory based on nanoindentation test results. The causes of mechanical properties deterioration are analyzed comprehensively from the aspects of microstructure and micromechanics.

## Experimental program

### Materials and sample preparation

The raw materials of concrete samples are Portland cement, sand, and coarse aggregate with black basalts. Table 1 listed the mix design used for the test samples. According to ASTM C192/C192M, the fresh concrete was cast in the oiled wood molds to form beams with dimensions of 3 × 4 × 16 in. Immediately after casting, all samples were externally vibrated for approximately 15 s and finished using a metal trowel. All specimens were demolded after 24 h and then cured in lime-saturated water at room temperature for at least 28 days. According to ASTM C666 (procedure A), which was designed to provide an indication of the potential freezing and thawing durability of concrete in a F–T environment, was adopted in this study. A total of nine beams were conditioned and they were then subjected to 0, 300, 500, 700, 900, and 1500 cycles, respectively.

**Table 1:** Existing mix design.

Cement(kg/m^3^)	Aggregate (kg/m^3^)	Sand (kg/m^3^)	Water/cement ratio
634.6	1076	753.5	0.48

### Nanoindentation investigation

**Figure 1:**
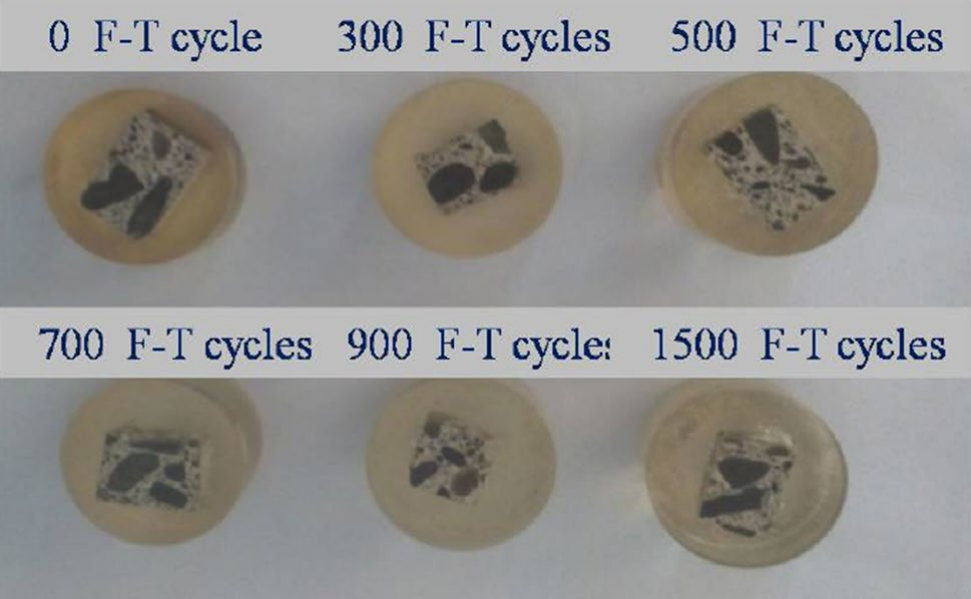
Polished samples with different F–T cycles^25^.

For the nanoindentation tests, six concrete groups of approximately 1.5 cm cubic specimens were cut from the beams after F–T cycles using diamond saws. Subsequently, the cubic specimens were put into the bottom of the plastic box, added the mixed solution of epoxy resin and curing agent, embedded in cylindrical shape. It is critical for obtaining more accurate indent test results to have a flat surface. Suitable grinding and polishing procedures are quite of importance. All cubic blocks were ground on 800, 1200, 2000 grit abrasive paper (Buehler) for approximate 3 min on grinding apparatus Buehler 250, respectively. Subsequently, they were polished with gradations 3 μm and 1 μm for about 3 min respectively, and 0.05 μm for 30 min. After grinding, the polished samples are shown in Fig. 1. For nanoindentaion test, of specimen with.

**Figure 2:**
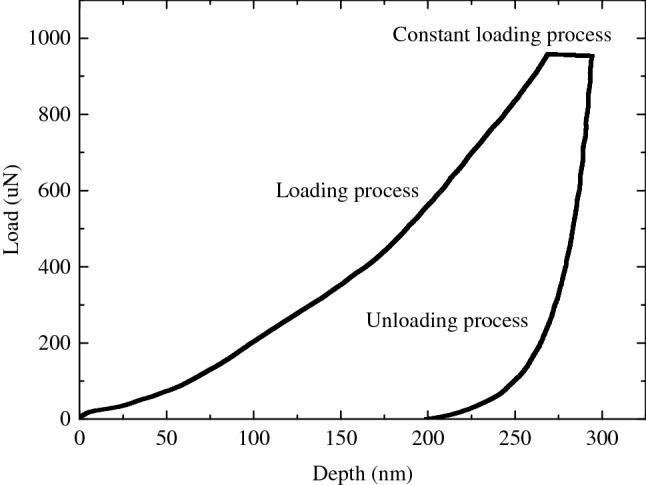
Load-depth curve of nanoindentation.

Firstly, the basic principle of nanoindentation is recalled briefly. During indentation, the indenter tip is pressed into the surface of tested material, force and displacement are recorded, and a load–displacement curve is generated subsequently (see Fig. 2). According to the curve, stiffness, hardness and indentation modulus can be derived by applying continuum scale models, respectively^26^.

Polished samples were examined using a Hysitron Ti 950 Triboindenter to probe micro-structural changes on different phases. A pyramid-shaped diamond Berkovich indenter with a tip radius of 100 nm and angle of 142.3° was employed to reduce the pile-up phenomena around the indent area in this study. Prior to test, Z-Axis calibration in the air, machine compliance, and tip area function calibration after each group of indentation were carried out in order to reduce the influence of instruments. In addition, indentation experiments were usually carried out at night in order to reduce the influence of thermal drift. Load-controlled indentation measurements were performed up to a maximum load of 2 mN at a loading rate of 0.2 mN/s followed by a hold time of 10 s, which can minimize creep effects^27^.

**Figure 3:**
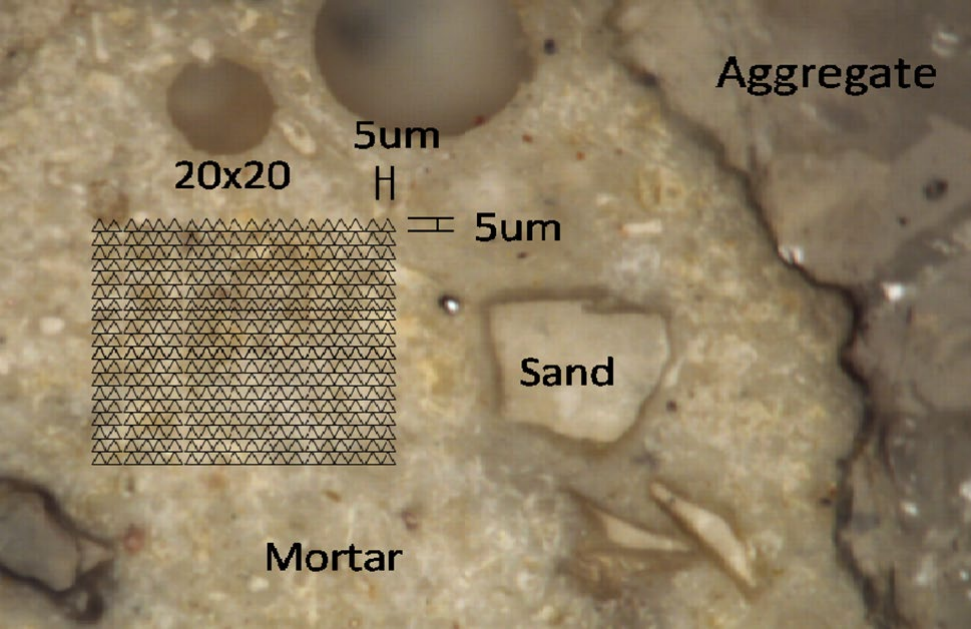
Indents in mortar surface.

**Figure 4:**
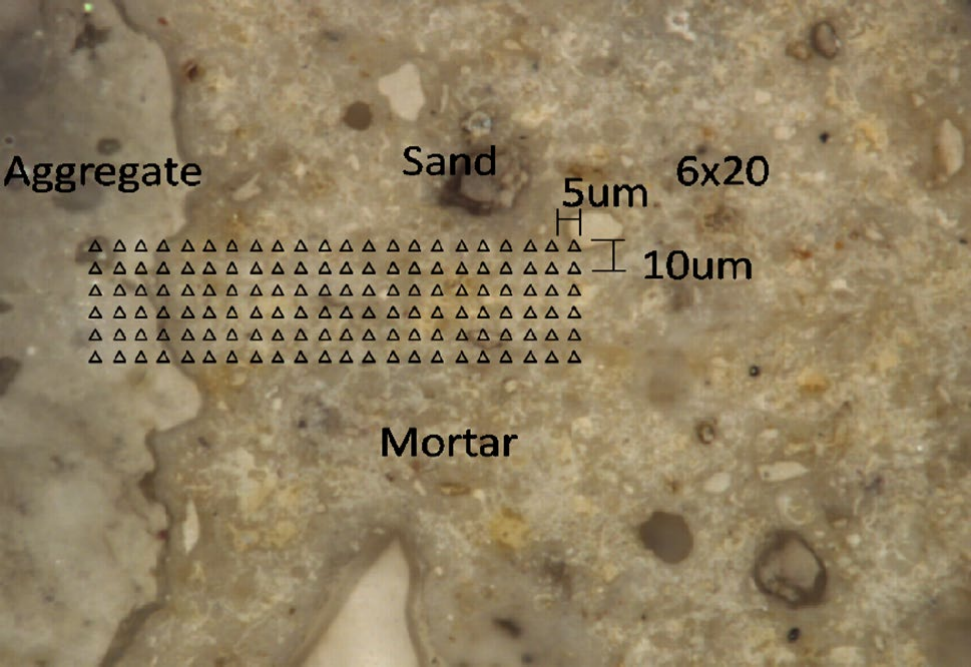
Indents in ITZ surface.

Since concrete is of highly heterogeneous nature in general, a large array of nanoindentation tests need to be carried out and analyzed statistically. To investigate the effect of different F–T cycles on the mechanical properties of concrete, each region consists of 400 indents with a spacing length of 5 μm is performed by randomly choosing the region on mortar surface (shown in Fig. 3). Secondly, a random indent region is chosen on the same aggregate, and the region consists of 120 indents with a spacing length of 5 um which is sufficiently large to avoid any interaction between two adjacent indentations^28^ to detect possible transition zone more comprehensive (see Fig. 4).

As for the treatment of indentation results, the statistical nanoindentation technique provides access to the properties of each phase. The volume fraction and mechanical properties of the individual active phases could be deconvoluted from a large number of nanoindentation tests^29^. Subsequently, a review of statistical nanoindentation is presented briefly. A large number of nanoindentation tests were performed on multiphase materials ($i = 1...Ni = 1...N$). It is assumed that the parameter distribution of each phase follows Gauss distribution:

1 $$\psi_{i} (x,\mu_{i} ,s_{i} ) = \frac{1}{{\sqrt {2\pi s_{i}^{2} } }}\exp (\frac{{ - (x - \mu_{i} )^{2} }}{{2s_{i}^{2} }})$$

where $u_{i}u_{i}$, $s_{i}s_{i}$ are the arithmetic means of all values of $jj$ phase and the standard deviation of the dispersion values respectively.

Thus, the probability density function of each phase is shown as:

2 $$P(x) = \sum\limits_{j = 1}^{n} {f_{j} } \psi_{j} (\mu_{j} ,s_{j} )$$

The volume fraction of phase *j* satisfies the condition:

3 $$\sum\limits_{j = 1}^{n} {f_{j} } = 1$$

Finally, through minimizing the difference between theoretical probability density and experimental probability density shown in Eq. (4), the mean and standard deviations of the mechanical parameters of each phase can be obtained:

4 $$\min \sum\limits_{j = 1}^{n} {\frac{{(P_{j} - P(x_{j} ))^{2} }}{n}}$$

where $P_{j}P_{j}$ is the observed value of the experimental frequency density.

### XRD investigation

X-ray diffraction analysis (XDR) could determine the mineral compositions and provide quantitative information for most of the crystalline phases^30,31^. To elucidate the evolution of mineral composition information of concrete, and more comprehensively verify the analysis results of nanoindentation test, XRD was used to characterize and quantify the mineral phases during F–T cycles, such as C-S–H and CH. Specimens were taken from the similar parts for the nannsoindentation tests. XRD equipped with Cu-Kα radiation was used to measure the powdered concrete samples, over a 2θ angular range from 5°to 120°.

## Experimental results and discuss

### Mechanical properties of mortar

**Figure 5:**
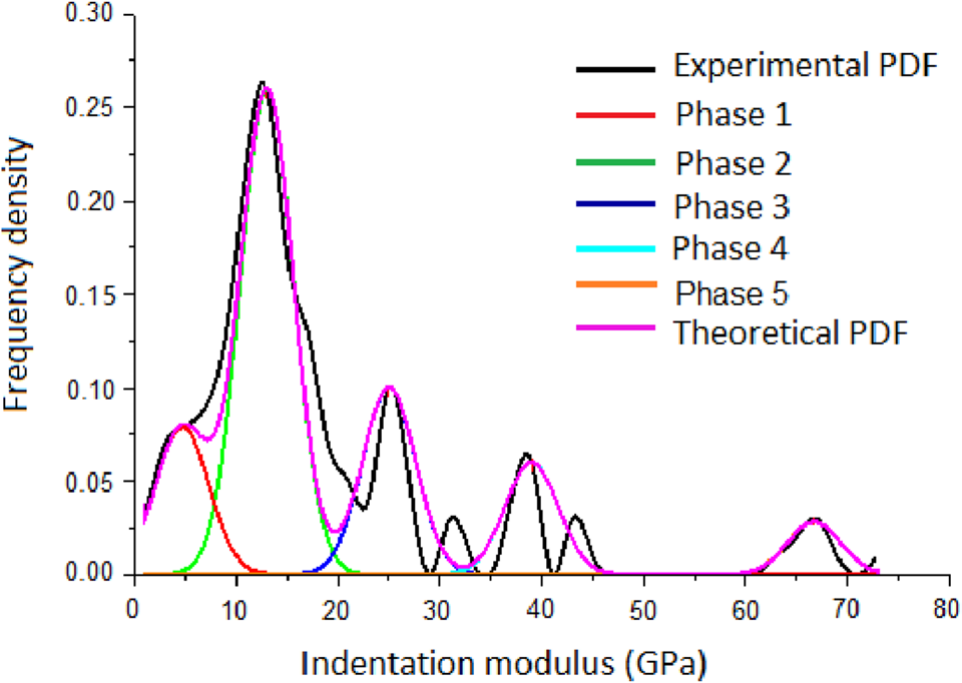
The frequency distribution of mortar indentation results.

**Figure 6:**
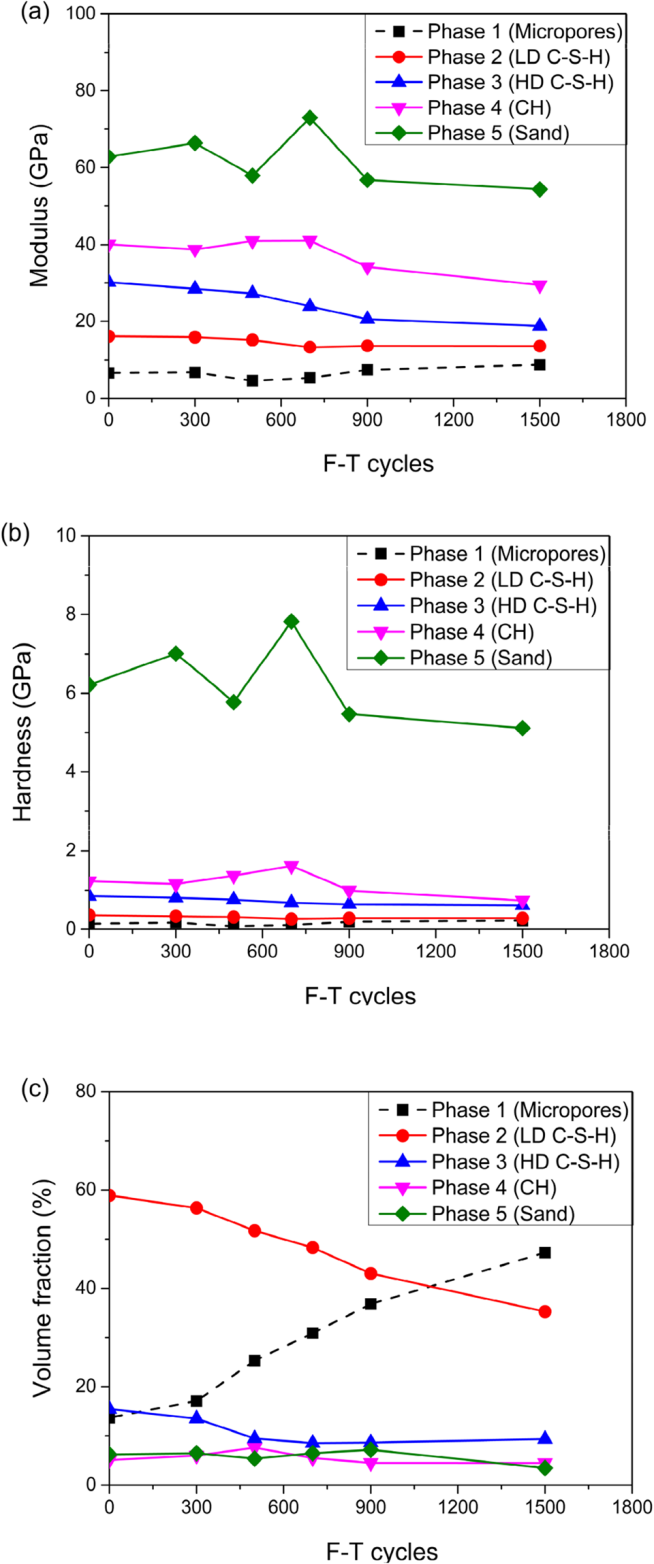
The deconvolution results of mortar.

**Figure 7:**
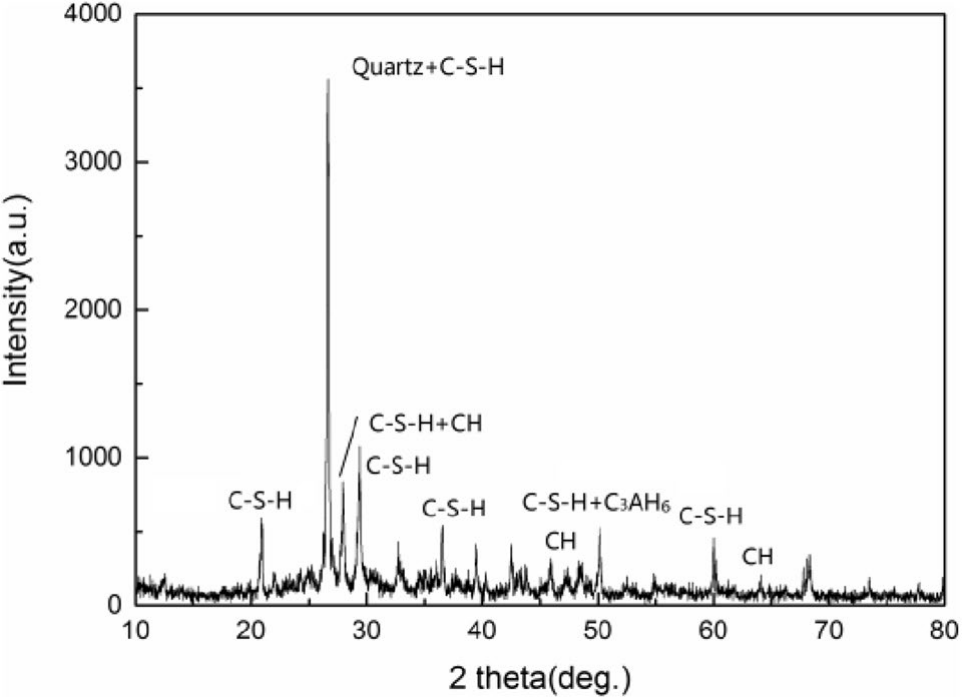
XRD test atlas of mortar.

The statistical analysis of the indent array on mortar and the deconvolution results are shown in Figs. 5 and 6, respectively. According to the cement mixture composition and hydration reaction, residual cement clinker is always present in cement-based materials with a water-to-cement ratio w/c < 0.42^32^, so the hydration products of the tested samples don't contain unhydrated cement clinker due to w/c = 0.48 of the tested samples. Table 2 summarizes the elastic moduli of the individual constituents found in either the open literature, or by experiments, which provide a reference to identify the nanoindentation results. Furthermore, the typical mineral compositions of the tested mortar under F–T cycles were investigated by XRD (see Fig. 7).The CH peaks at 2.63 Å and 4.90 Å were relatively few, and the principal calcium silicate hydrate identified were tobermorite (C_5_S_6_H_5_) with smaller amounts of xonotlite (C_6_S_6_H), dehydrated calcium aluminate hydrates (C_3_AH_6_) and quartz sand. The content of C-S–H and CH is approximately 15.7:1, which are in agreement with the results obtained by nanoindentation. Thus, it is reasonable to assume that the tested mortar compositions are mainly composed of micropores, low density calcium-

**Table 2:** Intrinsic elastic properties of cement paste constituents.

Constituent	Modulus(GPa)	Hardness(GPa)	Reference
CH	35.24	–	Beaudoin^33^
	40.3 ± 4.2	1.31 ± 0.23	Constantinides et al.^29^
LD C-S–H	18.8 ± 4	0.47 ± 0.17	Constantinides ^20^
	19.1 ± 5	0.66 ± 0.29	Dejong et al.^34^
HD C-S–H	29.4 ± 2.4	–	Constantinides et al. ^29^
	34.2 ± 5	1.36 ± 0.35	Sorelli et al. ^35^
Micropores	7 ± 4	0.19 ± 0.3	Sorelli et al.^35^
	9.1 ± 2.3	0.16 ± 0.07	Constantinides et al. ^29^
Unhydrated clinker	141.1 ± 34.8	9.12 ± 0.09	Sorelli et al. ^35^

silicate-hydrates (LD C-S–H), high density calcium-silicate-hydrates (HD C-S–H), calcium hydroxide (CH), and sand. Evidently, the two types of C-S–H are the major components of mortar with volume fraction of 58.9% and 15.45% respectively without F–T cycle and both the volume fraction of them have a certain downward trend with the increase of freeze–thaw cycles. The indent modulus of LD C-S–H, HD C-S–H, and CH in mortar decreased with the range of 15.9%, 37.7%, and 26.5% from 0 to 1500 F–T cycles respectively, while the hardness decreased with the range of 22.2%, 28.2%, and 39.3% from 0 to 1500 F–T cycles respectively.

**Figure 8:**
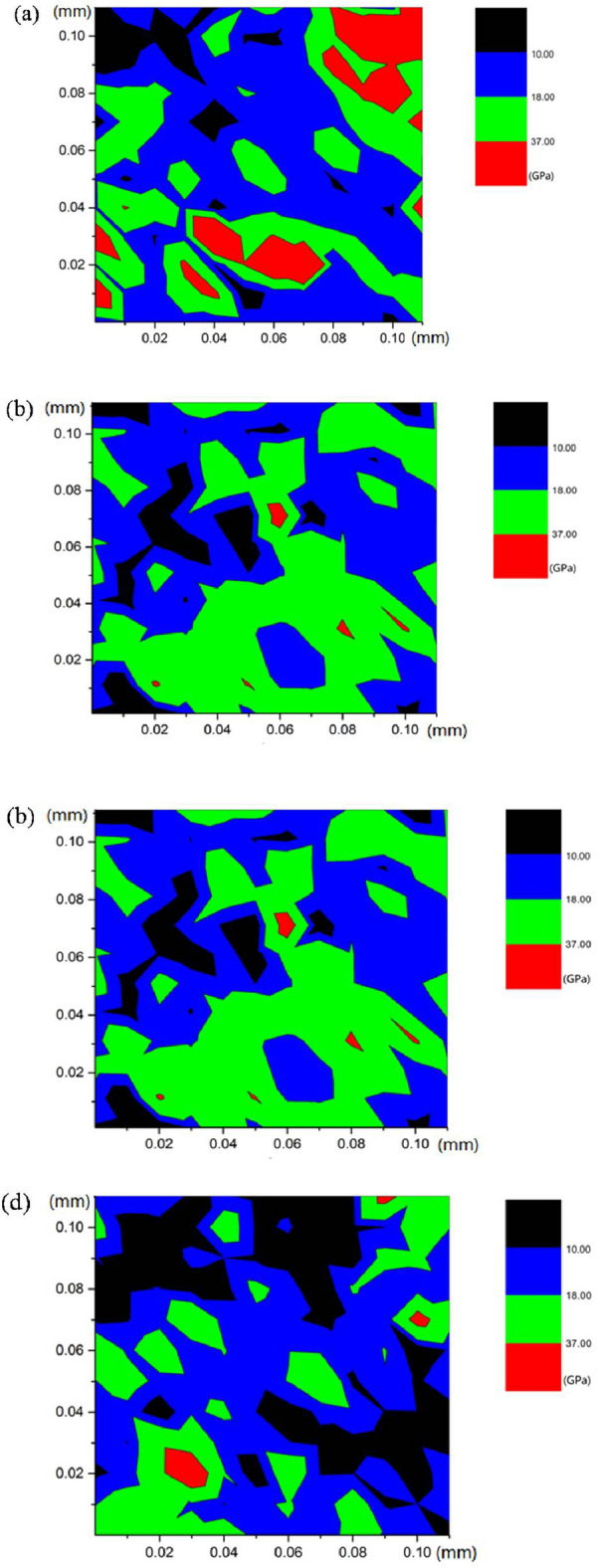
Contour map of nanoindentation results of mortar: (**a**) 0 cycle, (**b**) 300 cycles, (**c**) 700 cycles, (**d**) 1500 cycles.

The propagation of micropores under different F–T cycles is presented in Fig. 8 based on the contour map of nanoindentation modulus. Remarkably, the number of micropores in mortar increases gradually under 0 to 300 F–T cycles. Subsequently, the micropores gradually converge and connect to form larger diameter pores with the porosity reached 30.87% (see Fig. 8c,d) under 700 F–T cycles. Based on the above analysis, it could be concluded that the reduction of mortar under F–T condition is attributed to the degradation of modulus and hardness of microscopic components, and the increase and propagation of micropores.

### Mechanical properties of ITZ

**Figure 9:**
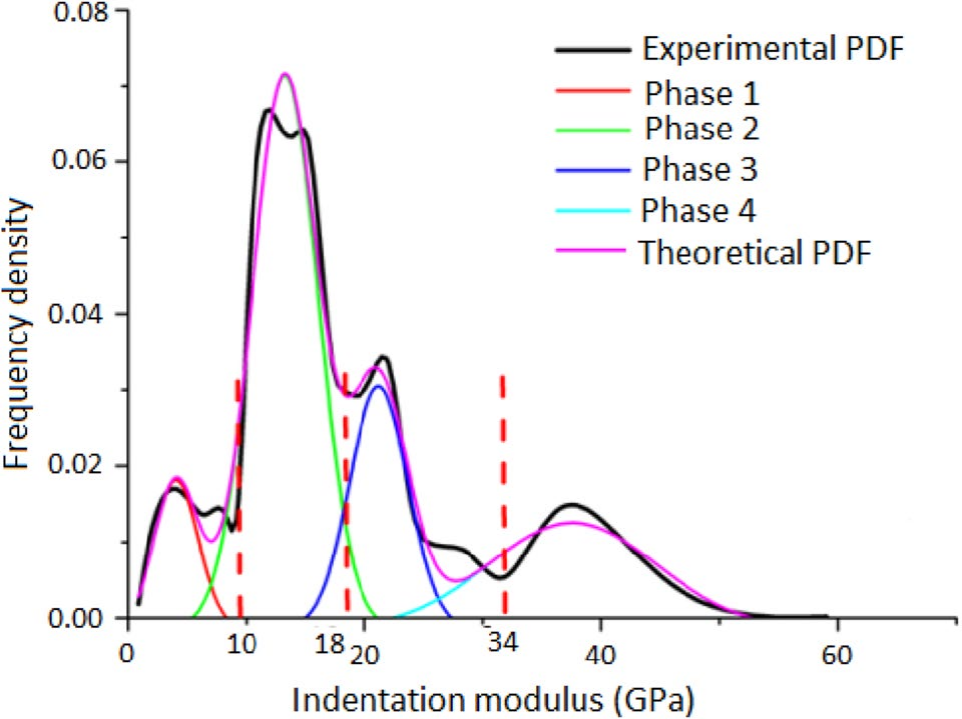
The frequency distribution of ITZ indentation results.

**Figure 10:**
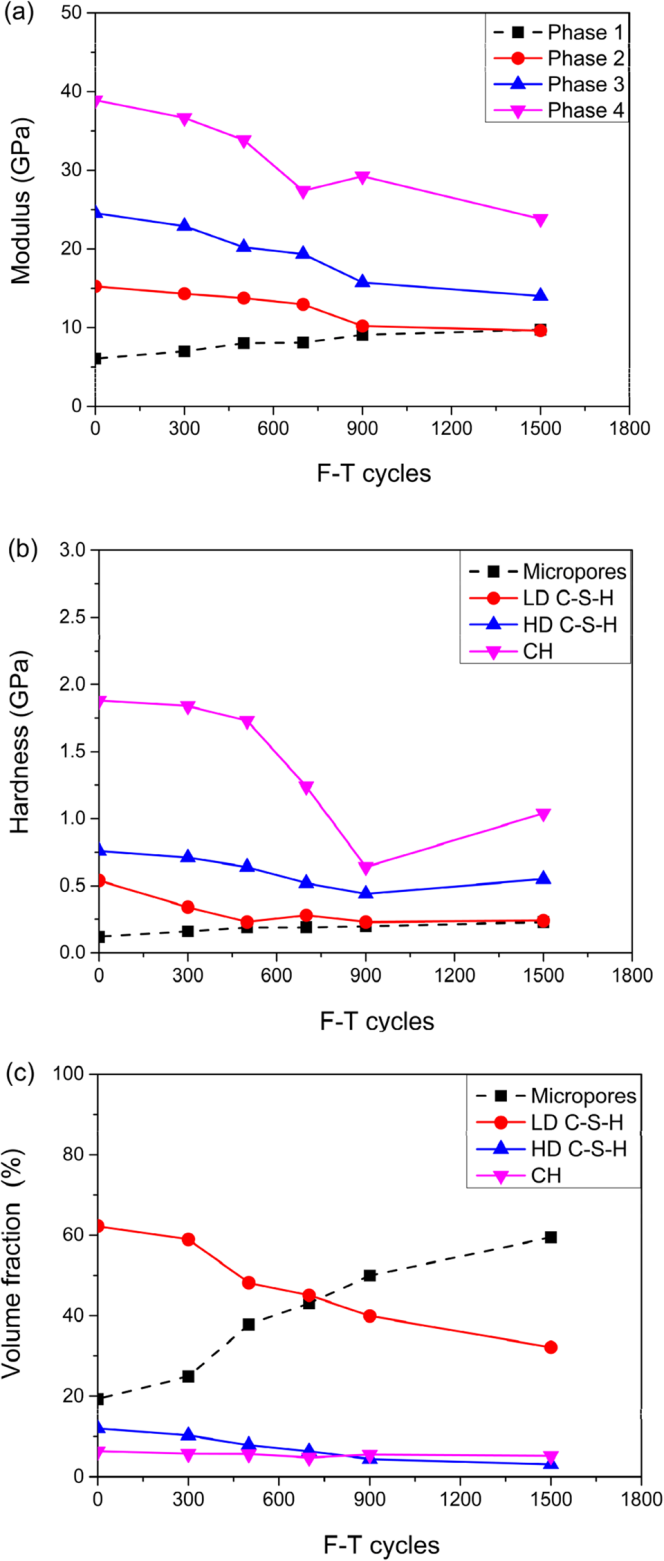
The deconvolution results of ITZ.

The statistical analysis of the indent array on ITZ and the deconvolution results are shown in Figs. 9 and 10, respectively. The two types of C-S–H are the major components of ITZ as before with the volume fraction of 62.29% and 12.03% respectively without F–T cycle. The modulus of elasticity of LD C-S–H, HD C-S–H, and CH in ITZ decreased with the range of 36.8%, 42.8%, and 38.7%, respectively, from 0 to 1500 F–T cycles while the hardness decreased with the range of 55.6%, 29.5%, and 44.7%, respectively from 0 to 1500 F–T cycles. Remarkably, the reduction range of modulus of elasticity and hardness of the above phases in ITZ is much more than that of the corresponding phases in mortar.

Noteworthy, the volume fraction of micropores increased rapidly to 59.52% after 1500 F–T cycles. The porosity of ITZ is much higher than that of mortar, and this is one of the main factors that result in the difference of mechanical properties between ITZ and mortar^36^.

**Figure 11:**
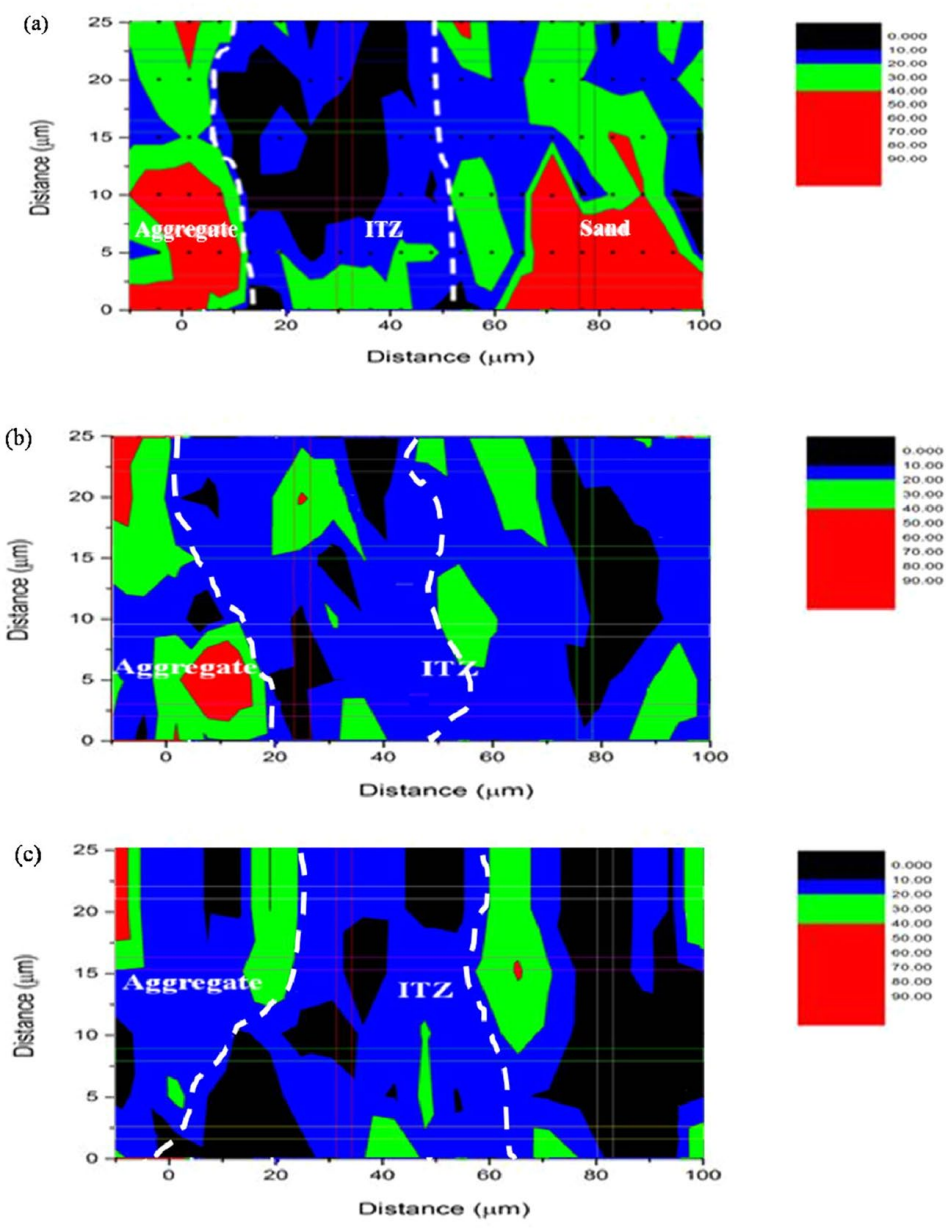
Contour map of ITZ thickness with 0, 700, and 1500 cycles^39^.

In the case of thickness of ITZ, a method based on contour map of nanoindentation results is adopted to determine the thickness in this paper. Figure 11a shows that there exists a weak band (the region between two dashed lines) with the average value of modulus less than 10 GPa around aggregate surface. Therefore, the weak band contains a large number of micropores. The thickness of ITZ is approximately 25 μm without F–T cycles, which is consistent to the thickness in literatures^37,38^. Subsequently, with the increase of F–T cycles, the number of micropores increases gradually, micropores converge and extend to larger pores gradually. The thickness of the weak band increases to 50 μm after 1500 F–T cycles rapidly (see Fig. 11b,c). Based on the above analysis, it could be concluded that F–T cycles has great impact on the micromechanical properties of ITZ. The rapid increase and propagation of micropores after F–T cycles results in the damage to ITZ.

## Multi-scale models for concrete

Concrete is a typical composite material with multiscale characteristics. The macroscopic performance of concrete depends on LD C-S–H at a nanoscale while it depends on mortar and coarse aggregate at a macroscale. The performance of each scale could be determined by the next one by one order of magnitude in size of the elementary heterogeneity. So far, the effective self-consistent scheme, asymptotic homogenization theory, Mori–Tanaka scheme and other schemes or methods have been extensively utilized to estimate the effective properties and failure characteristics of composite materials of the matrix-inclusion type^40,41^. For composites with complex structures, the derivation of the effective self-consistent scheme and asymptotic homogenization theory both are comparatively complex and requires a large amount of calculation, so the two schemes are rarely applied in the research of concrete performance^42^. Mori–Tanaka scheme has a simple and explicit structure, with clear physical explanations for all involved components. Meanwhile it is applicable for multiphase composites with various inclusion geometries, isotropy or anisotropies, and could have the good characterization of the multiple phases with the range of Poisson’s ratios considered (0.2 ~ 0.31)^32^, especially for concrete. In the study, Mori–Tanaka scheme is employed to estimate the effective modulus of elasticity of concrete subjected to F–T cycles. According to the scheme, the macroscopic strain **E** and the macroscopic stress of a representative elementary volume *V* could be determined by Eqs. (5) and (6), respectively^35^.

5 $$E = \frac{1}{V}\int {\varepsilon (x)} dV$$

6 $$\Sigma = \frac{1}{V}\int {\sigma (x)} dV$$

Through localization, the macroscopic strain and local strain could be obtained from Eq. (7). The The constitutive relation of local strain tensor and local stress tensor are determined from Eq. (8).

7 $$\varepsilon (x) = A(x):E$$

8 $$\sigma (x) = C_{r} :A(x):E$$

where $C_{r}$ represent the 4th-order stiffness tensor of the single phase.

If the representative elementary volume contains inclusions *r*, the homogenized stiffness tensor $\mathop C\nolimits^{\hom }$ follows from Eq. (9) through combining Eqs. (5) ~ (7).

9 $$\mathop C\nolimits^{\hom } = \frac{1}{V}\int {\mathop C\nolimits_{r} } :\mathop A\nolimits_{r} dV = \mathop C\nolimits_{0} + \sum\limits_{r}^{n} {\mathop f\nolimits_{r} } \frac{1}{V}\int {\mathop A\nolimits_{r} dV}$$

where *C*_0_, *f*_*r*_, and *A*_*r*_ are the stiffness tensor of the matrix, the volume fraction, and the 4th-order localization tensor of each inclusion, respectively.

The upscaling scheme is applied in four steps according to the order of magnitude in size of the elementary heterogeneity. The calculated results at each homogenization step of the multi-scale model are summarized in Tables 3 and 4.

**Table 3:** The homogenization step 1 and 2 of the multi-scale model.

Step 1				Step 2			
	*E* (GPa)	$\upsilon\upsilon$(1)	*f* (%)		*E* (GPa)	$\upsilon\upsilon$(1)	*f* (%)
LDC-S–H	15.5	0.24	79.2	C-S–H	17.6	0.23	79.8
HDC-S–H	29.2	0.24	20.8	Pores	0	0	14.7
				CH	40.1	0.3	5.5
Results	17.6	0.23			14.2	0.22

**Table 4:** The homogenization step 3 and 4 of the multi-scale model.

Step 3				Step 4			
	*E* (GPa)	$\upsilon\upsilon$(1)	*f* (%)		*E* (GPa)	$\upsilon\upsilon$(1)	*f* (%)
Paste	15.5	0.22	87.6	Mortar	14.1	0.23	73.7
Air voids	0	0	6.5	Aggregate	72.6	0.2	26.3
Sand	63.8	0.2	5.9				
Results	14.1	0.23			20.2	0.22	

**Figure 12:**
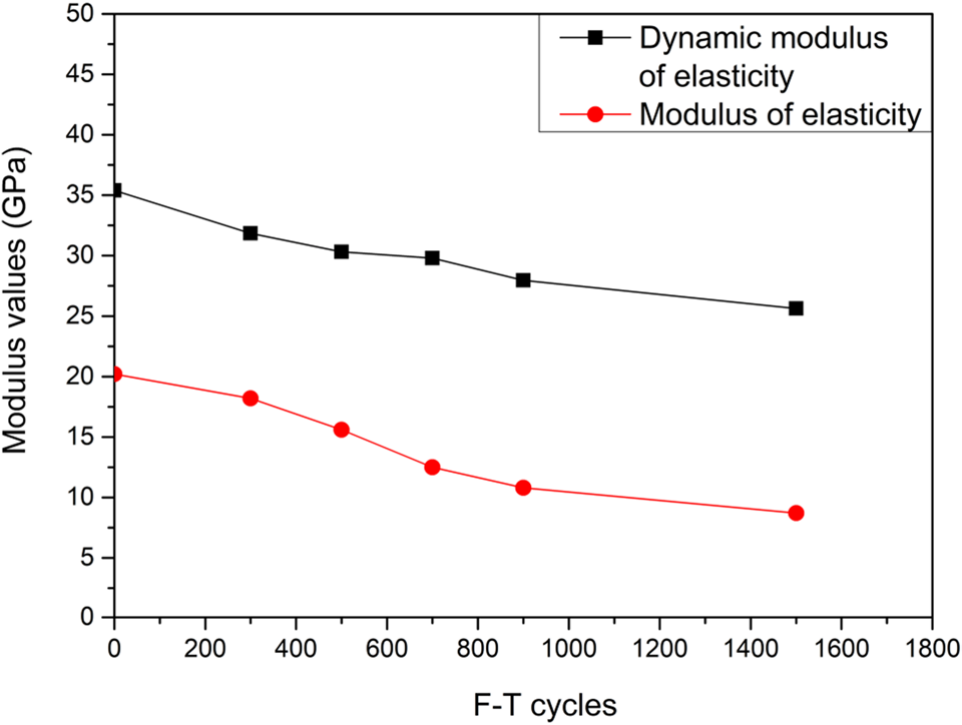
Modulus of concrete under F–T cycles.

The modulus of elasticity of concrete without F–T cycle measured from uniaxial loading test was 23.44 GPa while the calculated result by homogenization scheme was 20.2 GPa with the corresponding errors 13.2%. This calculated result is in agreement with the experimental value. Therefore, the modulus of elasticity of concrete at macroscale could be calculated using the homogenization scheme presented in this study. Due to the limited test conditions the modulus of elasticity of samples after F–T conditions were not conducted. The dynamic modulus of elasticity was higher than calculated modulus of elasticity; however, the dynamic modulus of elasticity has a linear correlation with modulus of elasticity^43,44^. Subsequently, in this research, the variation tendency of dynamic modulus of elasticity and the predicted modulus of elasticity under F–T cycles were compared. Figure 12 demonstrates the dynamic modulus of elasticity obtained by elastic wave method and static modulus of elasticity simulated through homogenization method under different F–T cycles respectively. It is shown that the dynamic modulus has a quickly decrease before 700 F–T cycles, and then the rate of descent is stabilize at approximate 25 GPa. For the calculated results, the rate of decrease is relatively smaller before 500 F–T cycles, and the rate of descent is in excellent agreement with that of experimental values. With the increase of F–T cycles, the thickness of ITZ increased, and then the micropores gradually extends to mortar, which has been verified through the results of nano CT scanning^45^. The number of micorpores in mortar increases rapidly, and the aperture size also expands after 700 F–T cycles. Therefore, it could be concluded that the mechanical properties deterioration of concrete is mainly attributed to the deterioration of mechanical properties (such as modulus and hardness) of microscopic components, and the increase and propagation of micropores especially for micropores in ITZ.

## Conclusions

In this paper, the micromechanical properties deterioration of concrete after 0, 300, 500, 700, 900, and 1500 F–T cycles were investigated by means of nanoindentation technique and XRD. The following conclusions could be drawn:

- 1. The indentation modulus and hardness of the main compositions in mortar (such as C-S–H and CH) both gradually decreases with the increase of F–T cycles, with the greatest reduction approximate 38% after 1500 F–T cycles. However, the more noteworthy is that the indentation modulus and hardness of the main compositions in interfacial transition zone (ITZ), show a more obvious decreasing tendency with the greatest reduction approximate 50%.
- 2. The volume fraction of miacropores in both mortar and ITZ increases as the F–T cycles increase. In addition, the micropores gradually converge and connect to form larger diameter pores with the porosity reaching 47.25% and 59.52% respectively under 1500 F–T cycles. The thickness of ITZ increased from 25 to 50 μm rapidly after 1500 F–T cycles.
- 3. Based on nanoindentation results, the effective modulus of elasticity under different F–T cycles are analyzed through Mori–Tanaka scheme. The calculated results correspond well with the test results and the variation tendency of dynamic modulus of elasticity.

The mechanical properties deterioration of concrete under F–T cycle is mainly attributed to the decrease of mechanical properties of microscopic components, and the increase and propagation of micropores especially for micropores in ITZ**.** It provided a helpful basis for finding effective measures of improving the F–T durability of concrete in engineering.

## Data Availability

Datasets are available in the manuscript. Any additional information and data are available upon reasonable request unless a small amount of data is not publicly available due to the commercial restrictions. The data and materials can be shared with corresponding author upon reasonable request.
